# Supplementary material for: miR-208b Reduces the Expression of Kcnj5 in a Cardiomyocyte Cell Line
Source: Biomedicines. 2021 Jun 23;9(7):719. doi: 10.3390/biomedicines9070719 (PMC8301481; doi:10.3390/biomedicines9070719)
Supplement: Supplementary file 1 [file biomedicines-09-00719-s001.zip › Supplementary Data miR-208b/Supplementary Data File 1.pdf]

Supplementary Data

miR-208b reduces the expression of Kcnj5 in a cardiomyocyte cell line

Julia Hupfeld[1#], Max Ernst[1#], Maria Knyrim[1#], Stephanie Binas[1], Udo Kloeckner[1], Sindy Rabe[1], Sigrid Mildenberger[1], Katja Quarch[1], Nicole Strätz[1], Danny Misiak[2], Matt Fuszard[3], Claudia Grossmann[1#], Michael Gekle[1#] and Barbara Schreier[1#*]

[1] Julius-Bernstein-Institute of Physiology, Medical Faculty of the Martin-Luther-University Halle-Wittenberg, Magdeburger Str. 6, 06110 Halle/Saale, Germany

[2] Institute of Molecular Medicine, Medical Faculty of the Martin-Luther-University Halle-Wittenberg, Charles Tanford Protein Center, Kurt-Mothes-Straße 3a, 06120 Halle (Saale), Germany

[3] Zentrum für medizinische Grundlagenforschung, Core Facility - Proteomic Mass Spectrometry, Proteinzentrum Charles Tanford, Martin-Luther-University Halle-Wittenberg, Kurt-Mothes-Straße 3a, 06120 Halle (Saale)

Max Ernst, Julia Hupfeld & Maria Knyrim should be considered joint first author. Barbara Schreier, Michael Gekle & Claudia Grossmann should be considered joint senior author.

*corresponding author: Barbara Schreier, Martin Luther University Halle-Wittenberg, Julius-Bernstein-Institute of Physiology, Magdeburger Str. 6, 06110 Halle/Saale, Germany, Tel: ++493455571886, Fax: ++493455574019, E-mail: barbara.schreier@medizin.uni-halle.de

Supplementary Table S1: Primers used for qPCR.

Gen	Protein	Ref. Seq.	Sense Primer (5'-3')	Antisense Primer(5'-3')	Annealing Temp [°C]	Product [bp]
18 S	-	NR_003278.3	GTAACCCGTTGAACCCCATT	CCATCCAATCGGTAGTAGCG	62	150
Gapdh	GAPDH	NM_002046.4	AAGGTGAAGGTCGGAGTCAA	AATGAAGGGGTCATTGATGG	60	107
Adra1a	adrenergic receptor, alpha 1a	NM_013461.4	CAGGGCCTCCGCAGC	AGTGACTCTCAACTTGGCCG	62	149
Cacnb2	L-typ Ca^{2+}-channel, subunit β2	NM_001252533.1	GCGAGGCAAAGCCACCTTTA	CTGTTCGTGCTGTAGCCTCA	60	492
Cacna1c	L-typ Ca^{2+}-channel, subunit α1	NM_009781.4	CCTGCTGGTGGTTAGCGTG	TCTGCCTCCGTCTGTTTAGAA	60	285
Kcnj5	GIRK4 (Kir3.4)	NM_010605.4	ATCTCCAGAAGTTAGCCCCAA	CATGCTCCCAAGTACACCCT	60	105
Kcnj2	potassium inwardly-rectifying channel, subfamily J, member 2	NM_008425.4	TCTCACTTGCTTCGGCTCAT	TGCTGTAGCGGTTGGTTCTC	61	372
Mapk10	mitogen-activated protein kinase 10	NM_009158.3	CGCTGTTGAGTTAAGACCCC	TCACATCCAAGGTTGGTTCA	60	107
Myh6	myosin, heavy polypeptide 6	NM_001164171.1	GCTGACAGATCGGGAGAATCAG	CCCCTATGGCTGCAATGC	64	121

Myh7	myosin, heavy polypeptide 7	NM_080728.2	GCATCAAGGAGCTCACCTACC	GGTAGCACAAGAGCTACTCCTC	63	285
Myh7b	myosin, heavy chain 7B	NM_001085378.2	ACTCAAGCGGGAGAACAAGA	ACCTGGGACAACTCCAACTG	64	213
Ppm1k	protein phosphatase 1K	NM_175523.4	ACTGGTGGTAGCCAGTGTTG	CTGTCGATCAGGCCCATCTC	60	527
RGS2	regulator of G-protein signaling 2	NM_009061	TGCGTACCCATGGACAAGAG	GCCTCTCCTAGGTTGCTGAC	62	783
Slc25a22	solute carrier family 25, member 22 (Slc25a22)	NM_026646.3	GAGGGCTACTTCGGCATGTA	AGGTCAGCTTCTGTCCATCC	62	570
SOD2	superoxide dismutase 2, mitochondrial	NM_013671.3	AGGAGAGTTGCTGGAGGCTA	TAGTAAGCGTGCTCCCACAC	61	228

Supplementary table S2: Taqman™ assays for the detection of miRNAs and pri-miRNAs

Taqman Assay	Order ID	miRBase Accession Nr
hsa-miR-208b	002290	MIMAT0004960
hsa-miR-208	000511	MIMAT0000241
hsa-miR-449c *	241086_mat	MIMAT0010251
pri-miR-208b	Mm03308667_pri	
U6 snRNA	001973	

Supplementary Table S3: Dual Luciferase reportergene constructs obtained from GeneCopoeia containing the 3’-UTRs of the L-type calcium channel subunits with the respective RefSeq gene accession number.

Reporter construct	Length 3´-UTR	RefSeq
pEZX-MT06-Cacnb2	1832 bp	NM_001252533.1
pEZX-MT06-Cacna1c_a	2228 bp (-16 - 2211 bp)	NM_001159533.2
pEZX-MT06-Cacna1c_b	2322 bp (2143 - 4464 bp)	NM_001159533.2
pEZX-MT06-Cacna1c_c	2299 bp (4393 - 6691 bp)	NM_001159533.2
pEZX-MT06-Kcnj5	2797 bp	NM_010605.4
pEZX-MT06-SOD2	2947 bp	NM_013671.3

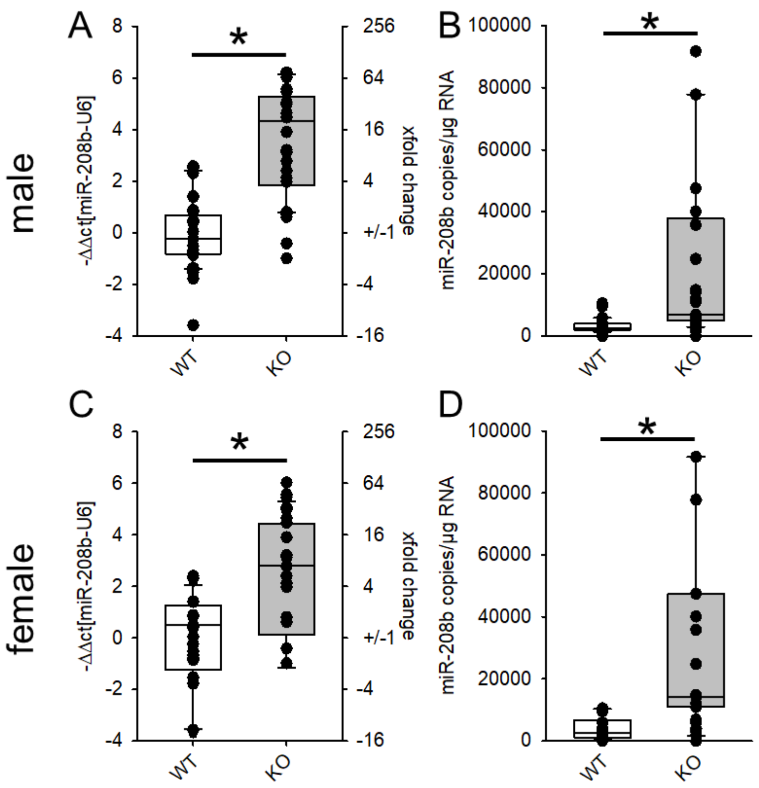

Supplementary Figure S1. Expression of miR-208b in whole hearts from mice with severe heart hypertrophy. Realtime qRT-PCR of (**A**) male (N=10 animals/group) and (**C**) female (N= 8-10 animals/group) or (**B**) digital droplet PCR of male (N=10 animals/group) and (**D**) female (N= 8-10 animals/group) mice.

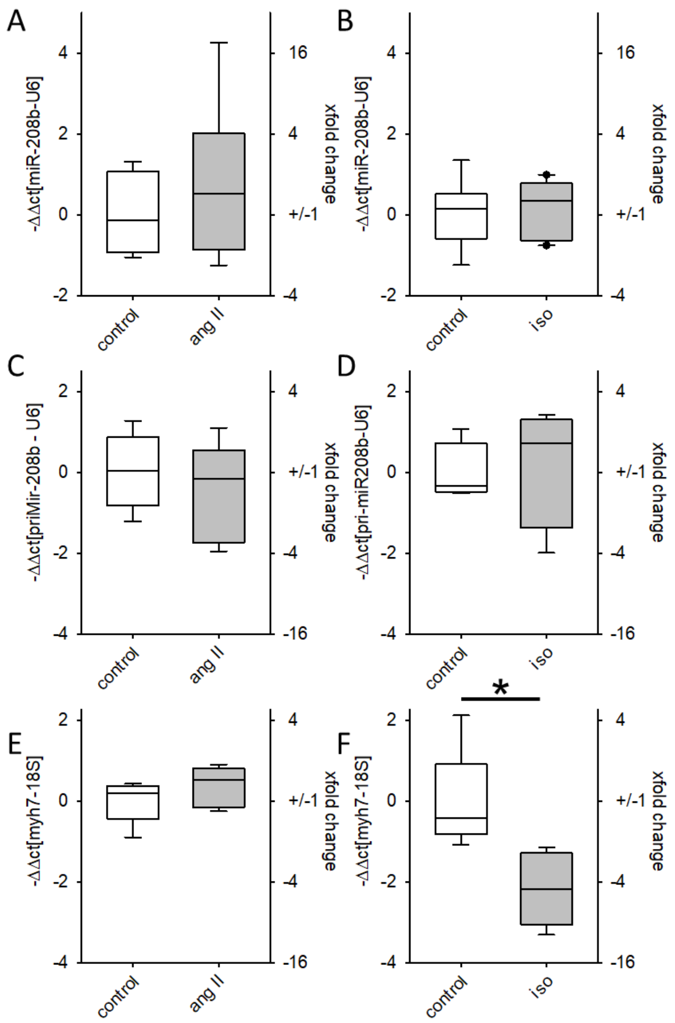

Supplementary Figure S2: Expression of miR-208b (A, B), pri-miR-208b (C,D) or myh7 (E, F) in whole heart samples from mice treated with AII (N= 5-7 animals/group) or isoprenaline (N= 5 animals/group).

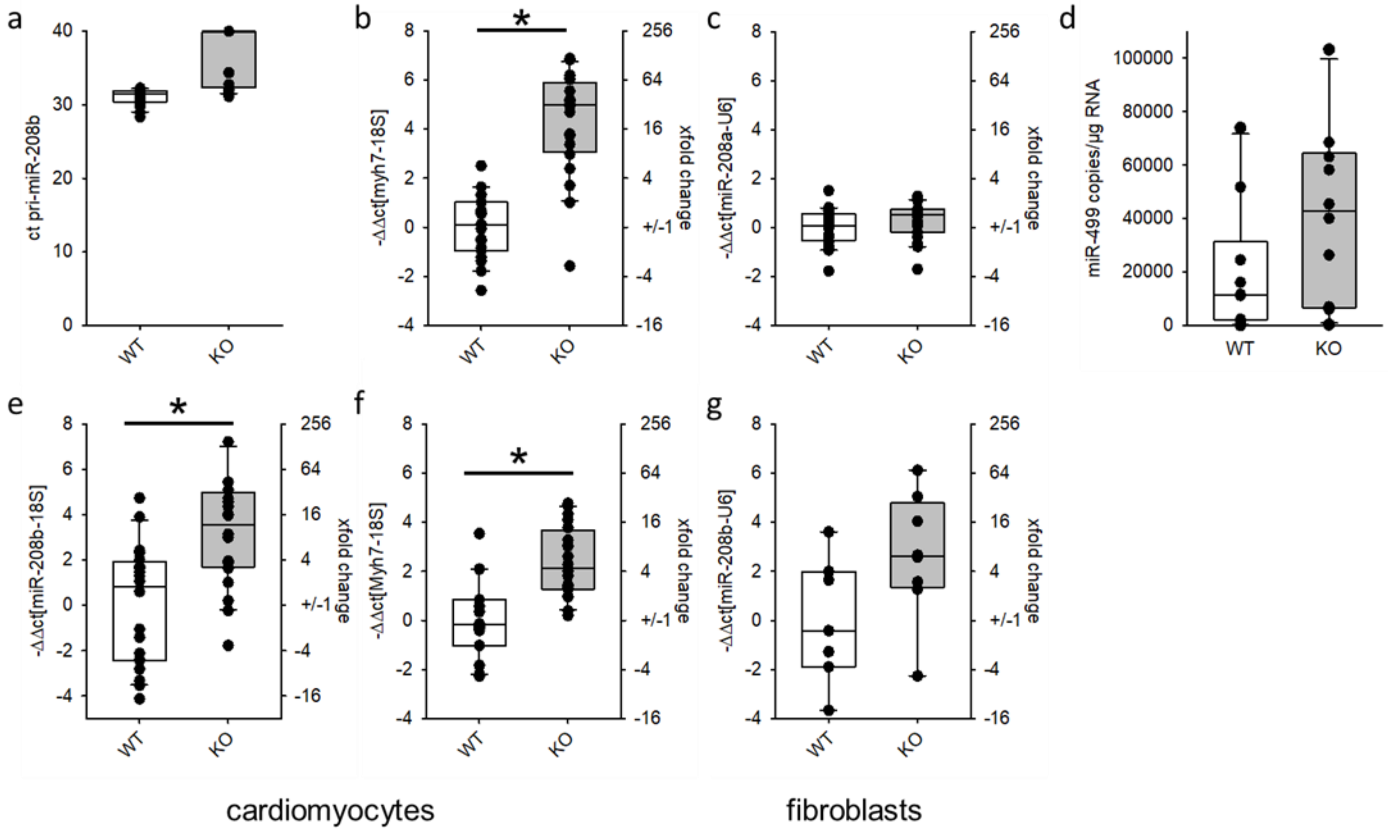

Supplementary Figure S3. (**a**) Expression (real-time qRT-PCR) of pri-miR-208b in whole heart samples from male and female KO animals (N= 19-20 animals/group). (**b**) Expression (real-time qRT-PCR) of the miR-208b host gene myh7 in whole heart samples of mice with severe heart hypertrophy (N = 19-20 animals/group). (**c**) Expression (real-time qRT-PCR) of miR-208a in whole heart samples of mice with severe heart hypertrophy (N= 19-20 animals/group). (**d**) Expression (digital droplet PCR) of miR-499 in heart samples of mice with severe heart hypertrophy (N=10 animals/group). (**e**) Expression (real-time qRT-PCR) of miR-208b in isolated, adult cardiomyocytes from mice with severe heart hypertrophy (N=20 animals/group). (**f**) Expression (real-time qRT-PCR) of myh7 in isolated, adult cardiomyocytes from mice with severe heart hypertrophy (N=20 animals/group). (**g**) Expression (real-time qRT-PCR) of miR-208b in isolated, cardiac fibroblasts from mice with severe heart hypertrophy (N=7-8 animals/group).

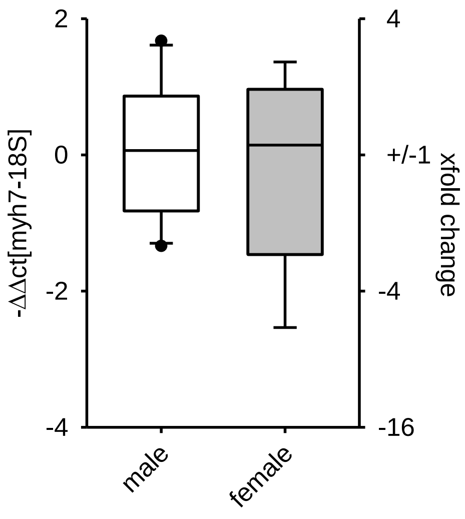

Supplementary figure S4: Expression of myh7 in whole heart samples from male and female untreated wildtype animals (N = 9-10 animals/group)

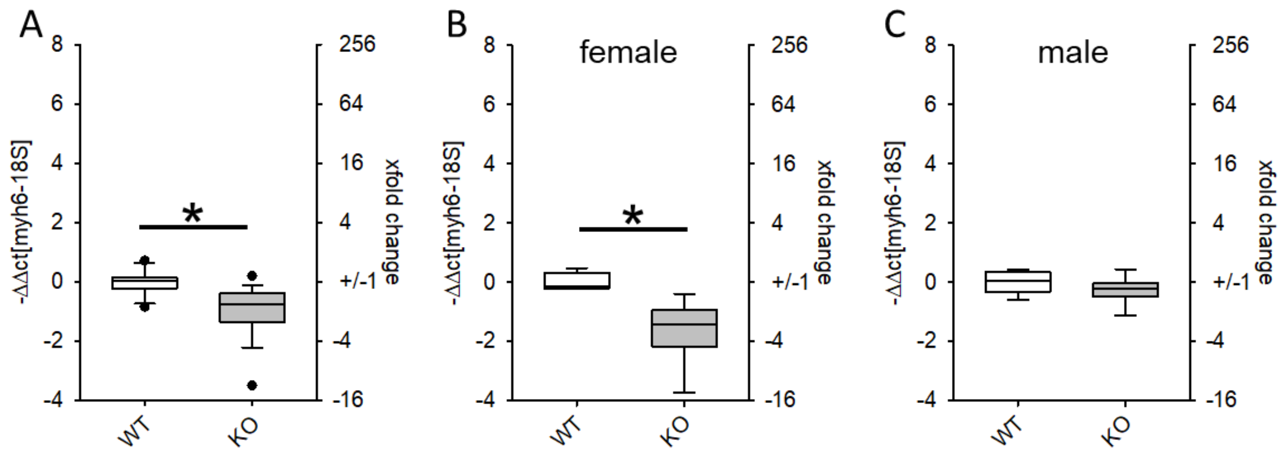

Supplementary figure S5: Expression of myh-6 in whole heart samples from adult mice with severe heart hypertrophy in both gender (A, N = 18 animals/group), in females (B, N = 9 animals/group) or in males (C, N = 9 animals/group).

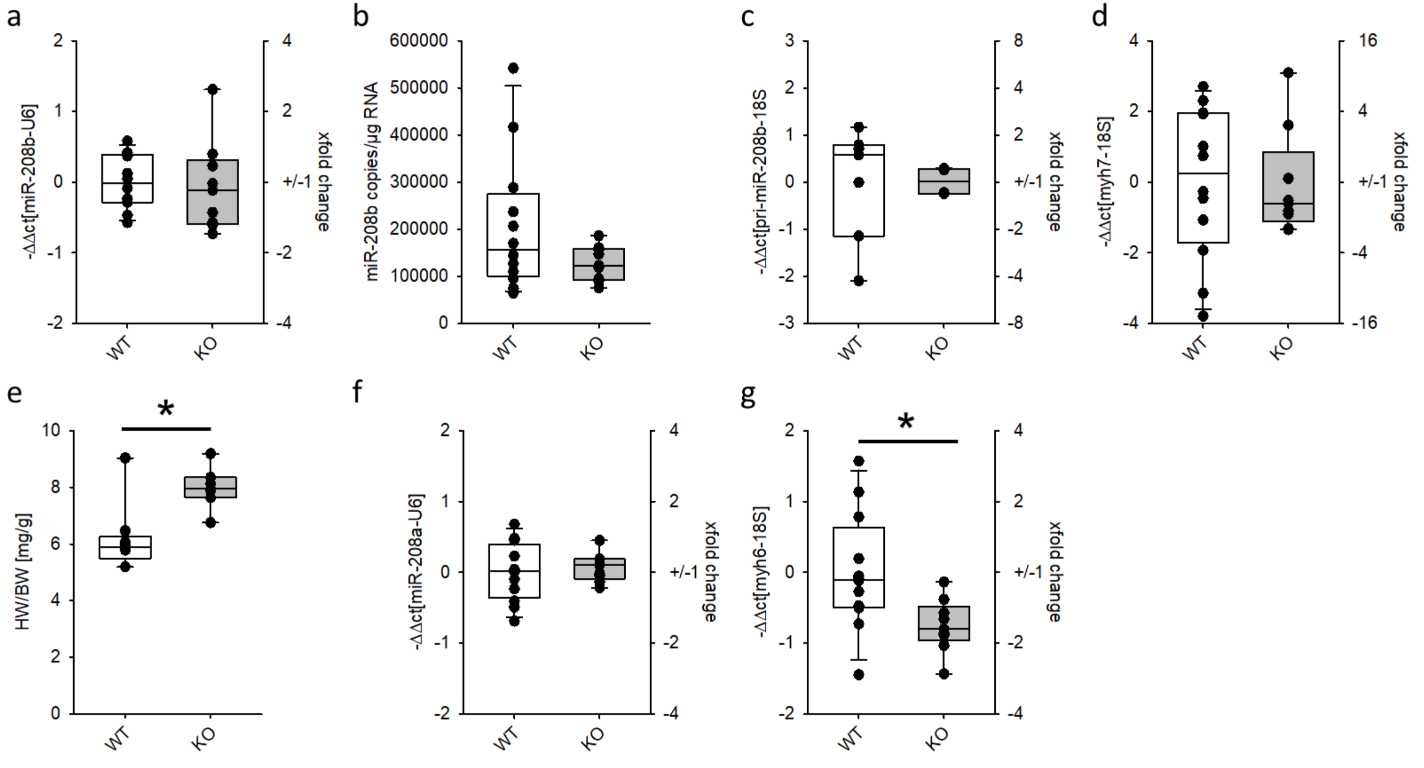

Supplementary Figure S6: Gene expression in whole heart samples from newborn (1 week) KO and WT animals. (a) Real-time qRT-PCR for miR-208b (N= 19-20 animals/group), (b) digital droplet PCR of miR-208b (N= 19-20 animals/group), (c) real-time qRT-PCR for pri-miR-208b (N= 4-7 animals/group), (d) real-time qRT-PCR for myh7 (N= 9-12 animals/group), (e) heart weight to body weight (HW/BW) ratio (N= 7-9 animals/group), (f) real-time qRT-PCR for miR-208a (N= 5-7 animals/group), (g) real-time qRT-PCR for myh6 (N= 9-12 animals/group).

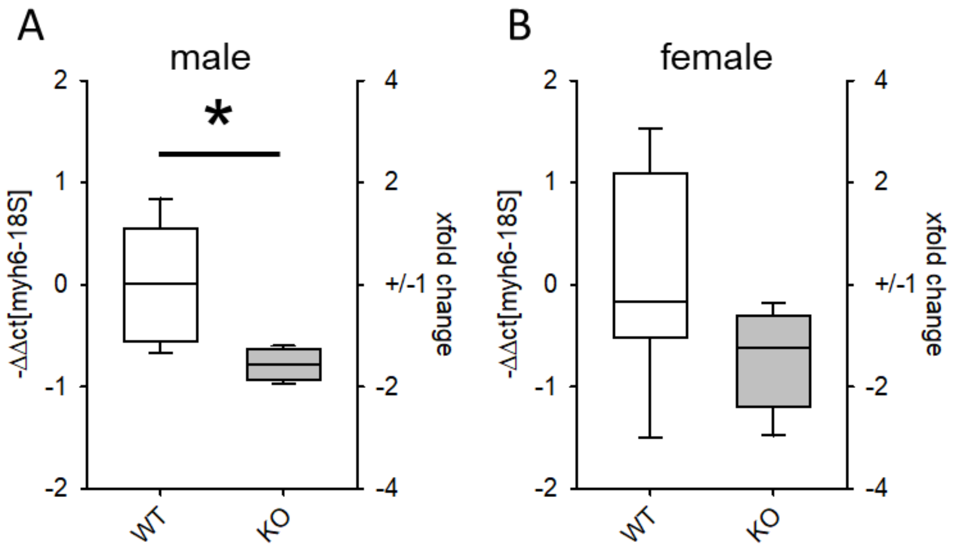

Supplementary figure S7: Expression of myh6 in whole heart samples of mice 1 week after birth discriminated between male (A, N = 4-5 animals/group) and female (B, N = 5-7 animals/group) animals.

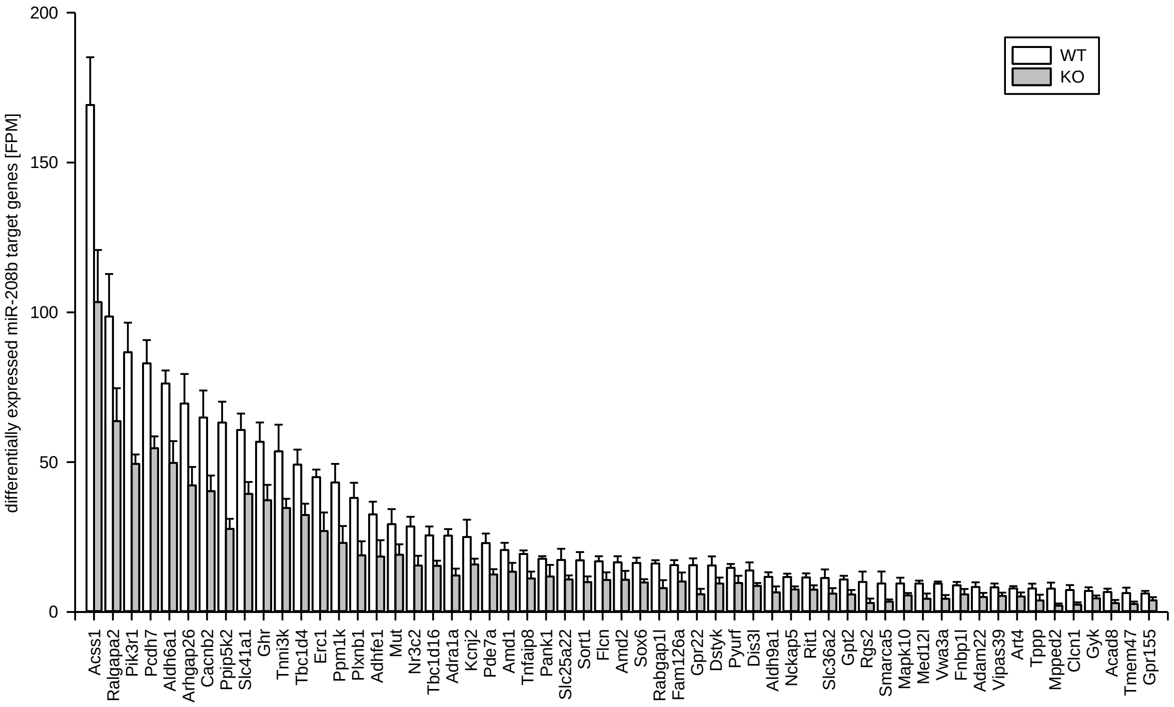

Supplementary Figure S8: Potential target genes of miR-208b identified by comparison of next generation sequencing data from whole heart samples of mice with severe heart hypertrophy to 3’-UTR predicted target genes by miR-walk 2.0 (N= 6 animals/group)

Cohen D ≥│1.5│, FC ≥│1.5│		
	upregulated	downregulated
miR-208b mimic	18	22

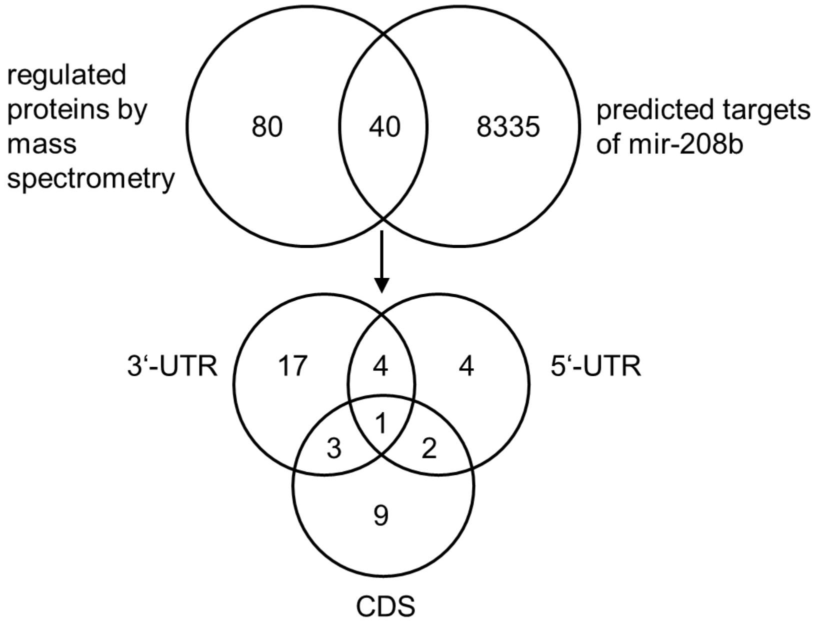

Supplementary Figure S9: HL-1 cells were transfected with miR-208b mimics or mimic control. Protein expression was analyzed with mass spectrometry (N = 6 samples/group).

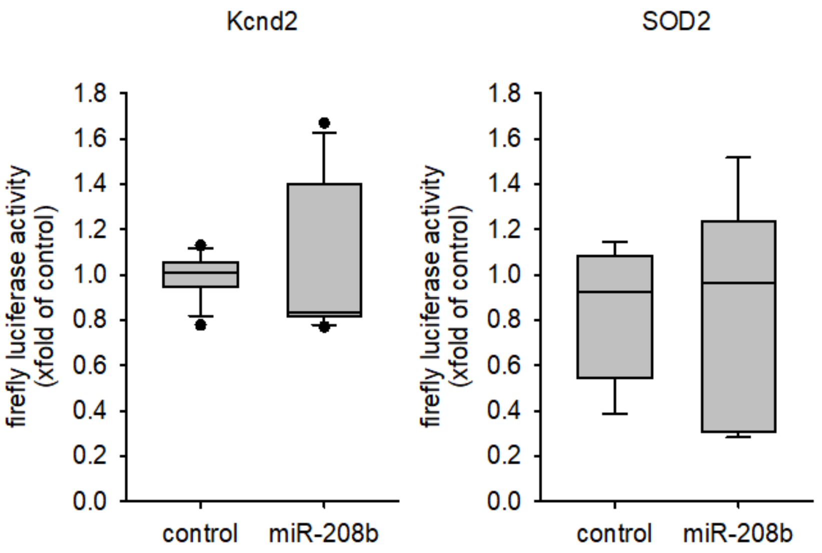

Supplementary figure S10: To evaluate if the miRNAs bind to the 3’-UTR dual luciferase constructs containing the 3’-UTR from the potassium voltage-gated channel subfamily D Member 2 (Kcnd2) or the superoxide dismutase 2 (SOD2), the seed sequence or an empty vector was transfected in HEK293 cells either with or without mimic control or miR-208b mimic. miR-208b mimic reduced the luciferase activity of the Cacna1c-II and the Kcnj5 construct (N=3-4 experiments/group, n = 9-12 wells/group).

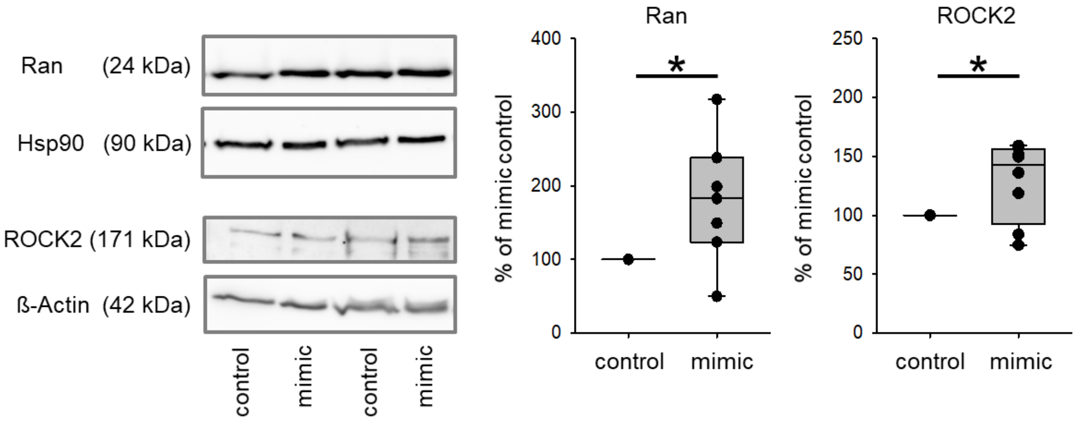

Supplementary Figure S11: Western blot analysis for Ran and Rock2 was performed in HL-1 cells treated either with mimic control or miR-208b mimics ($N = 8$ experiments/group). Representative Western blots are given.
